# Effects of oral and transdermal estrogen on postoperative endometrial recovery after missed abortion: a retrospective longitudinal cohort study

**DOI:** 10.3389/fmed.2025.1713144

**Published:** 2025-12-03

**Authors:** Lushuang Zhang, Shiyang Liu, Yunyi Su, Liubiqi Zhao, Xiaoqin Gan, Xuan Duan

**Affiliations:** Department of Gynecology and Obstetrics, Chengdu Women’s and Children’s Central Hospital, School of Medicine, University of Electronic Science and Technology of China, Chengdu, China

**Keywords:** missed abortion, estrogen therapy, oral estrogen, transdermal estrogen, cohort study

## Abstract

**Background:**

Missed abortion is a common complication of early pregnancy, and surgical evacuation remains the primary treatment. This study aims to evaluate the effects of no estrogen, oral estrogen, and transdermal estrogen on postoperative endometrial recovery and long-term reproductive outcomes.

**Methods:**

This retrospective longitudinal cohort study included women with early missed abortion who underwent surgical evacuation at Chengdu Women’s and Children’s Central Hospital between June 2023 and May 2025. Patients were stratified into three groups: no-estrogen, oral estrogen, and transdermal estrogen. Clinical data were collected at five time points: perioperative period, 1 week postoperatively, 1 month postoperatively, 3 months postoperatively, and 1 year postoperatively. Primary outcomes were endometrial thickness, intrauterine adhesion, adverse reactions, and 1-year pregnancy outcomes. Statistical analyses comprised the *t*-test, the chi-square test, the binary logistic regression analysis, and the multiple linear regression analysis.

**Results:**

A total of 561 women were included. Endometrial thickness at 1 and 3 months was significantly greater in both estrogen groups compared with the no-estrogen group (*p* < 0.001 and *p* = 0.001, respectively), with the transdermal group showing the highest values. Reduced menstrual flow was less frequent in the estrogen groups (*p* = 0.022). Oral estrogen was associated with higher rates of breast tenderness and systemic symptoms, whereas transdermal estrogen demonstrated superior adherence (68.3% vs. 46.7%, *p* < 0.001). At 1-year follow-up, pregnancy outcomes did not differ significantly across groups (*p* = 0.819), although higher conception rates were observed in the estrogen groups, particularly among those receiving transdermal therapy.

**Conclusion:**

Postoperative estrogen therapy promotes endometrial recovery after the surgical treatment of missed abortion. Both oral and transdermal estrogen treatments were effective, but transdermal administration provided better tolerability and adherence. Estrogen use may improve reproductive prognosis, warranting further prospective studies to confirm these findings.

## Background

Missed abortion, also referred to as early pregnancy loss with retained gestational tissue, is one of the most frequent complications in early pregnancy (1, 2). It is typically diagnosed by ultrasound when embryonic or gestational structures fail to demonstrate the expected development or cardiac activity (3). The prevalence of missed abortion has been rising globally, largely attributable to delayed childbearing, increased exposure to environmental risk factors, and the growing burden of reproductive disorders (4, 5). Beyond its clinical implications, missed abortion often imposes profound psychological stress and emotional trauma on affected women and their families.

Surgical evacuation remains the primary treatment for missed abortion because of its rapid effectiveness and widespread availability (6, 7). Nevertheless, surgical procedures may cause direct damage to the endometrium, resulting in intrauterine adhesions, impaired endometrial receptivity, abnormal menstruation, and, in severe cases, secondary infertility. Given the importance of preserving fertility, enhancing postoperative endometrial repair, and minimizing adverse reproductive sequelae are critical goals in the management of women undergoing surgical evacuation (8, 9).

Hormonal therapy, particularly estrogen supplementation, has long been recognized as an effective strategy to stimulate endometrial proliferation, improve vascularization, and reduce adhesion formation (10). However, the optimal route of estrogen administration remains controversial. Oral estrogen, although effective, undergoes first-pass hepatic metabolism and may be associated with systemic side effects and reduced adherence (11). In contrast, transdermal estrogen bypasses hepatic metabolism, providing more stable plasma concentrations and potentially fewer adverse effects. Despite these pharmacological differences, comparative clinical evidence regarding their effectiveness in postoperative endometrial recovery and long-term reproductive outcomes is still limited (12).

Existing studies have primarily focused on short-term outcomes, such as endometrial thickness and menstrual restoration, often neglecting long-term reproductive endpoints, including conception rates, miscarriage risk, and live birth outcomes (13, 14). Furthermore, a majority of studies are cross-sectional or short-term interventional trials, with limited data from longitudinal real-world cohorts. This lack of comprehensive evidence hampers the development of standardized clinical guidelines for the postoperative management of missed abortion.

To address these gaps, the present retrospective longitudinal cohort study was conducted to evaluate the effects of no-estrogen, oral estrogen, and transdermal estrogen on postoperative endometrial recovery and long-term pregnancy outcomes in women with missed abortion. By integrating perioperative clinical characteristics with short- and long-term follow-up data, this study provides new insights into optimizing postoperative hormonal therapy strategies and improving reproductive prognosis in this vulnerable patient population.

## Methods

### Study population

This retrospective longitudinal cohort study is part of the Longitudinal Missed Abortion Study (LoMAS) conducted in Chengdu (China Clinical Trials Registry, ChiCTR2200059282) and was approved by the Ethics Committee of Chengdu Women and Children’s Central Hospital (No. 2022207). From June 2023 to May 2025, women diagnosed with early missed abortion and treated with surgical evacuation at Chengdu Women’s and Children’s Central Hospital were consecutively included. The ultrasound diagnostic criteria for early missed abortion were as follows: crown–rump length ≥ 7 mm with absent fetal cardiac activity; mean gestational sac diameter ≥ 25 mm with no embryo; intrauterine gestational sac without yolk sac and no embryo or cardiac activity observed after 2 weeks; and intrauterine gestational sac with yolk sac but no cardiac activity after 11 days.

Inclusion criteria were as follows: age 25–45 years; fulfillment of the diagnostic criteria for early missed abortion; normal liver and renal function and normal coagulation parameters; amenorrhea duration of 8–12 weeks; and normal ovarian function. Exclusion criteria were as follows: allergy to estrogen or alcohol; concomitant benign or malignant tumors or other reproductive system diseases; breast diseases, including breast tumors or hyperplasia; impaired consciousness or cognitive dysfunction; risk of thrombosis or history of venous thromboembolism; and incomplete baseline information or poor treatment compliance. The sample size was estimated based on previous studies evaluating the effects of estrogen on endometrial thickness after miscarriage (15), resulting in a minimum requirement of 87 participants per group. In this study, all eligible cases within the study period were included, and the final sample size exceeded this minimum requirement.

### Interventions

Patients who met the eligibility criteria were retrospectively categorized according to the postoperative hormonal regimen received. Oral estrogen group: oral estradiol valerate 1 mg (Progynova, DELPHARM Lille S.A.S, Import Drug Registration No. H20160679, Chinese approval No. J20171038) was administered daily for 24 days. From day 15, dydrogesterone 10 mg (Duphaston, Abbott Healthcare Products B.V, Import Drug Registration No. H20170221) was administered daily for 10 days. Transdermal estrogen group: transdermal estradiol gel was administered once daily for 24 days. From day 15, dydrogesterone 10 mg was administered daily for 10 days. No-estrogen group: patients who did not receive any estrogen therapy postoperatively. For both estrogen-treated groups, the regimen was continued for three consecutive cycles, with each cycle lasting 1 month.

### Data collection

Clinical data were collected at five longitudinal time points: perioperative period, 1 week postoperatively, 1 month postoperatively, 3 months postoperatively, and 1 year postoperatively. Information included age, occupation, work intensity, educational level, menstrual history, obstetric history, sexual history, body mass index, surgical history, comorbidities, medication history, smoking and alcohol use, hygiene habits, family history, use of postoperative analgesics, length of hospital stay, and perioperative complications (fever, abdominal pain, heavy vaginal bleeding, or pulmonary infection). Long-term reproductive outcomes included subsequent conception, biochemical pregnancy, missed abortion, threatened abortion, assisted reproductive technology use, and ectopic pregnancy.

Perioperative data were extracted from the hospital electronic medical records system. Follow-up data at 1 week, 1 month, and 3 months were collected through outpatient visits, while 1-year pregnancy outcomes were obtained via telephonic follow-up. Primary outcomes included postoperative endometrial thickness (day 20), duration of vaginal bleeding, time to menstrual resumption, rate of reduced menstrual flow, serum estrogen levels, incidence of intrauterine adhesion (at 3 months), adverse reactions, and 1-year pregnancy outcomes.

### Statistical analysis

All analyses were performed using SPSS version 25.0 (IBM Corp., Armonk, NY, United States). Continuous variables with normal distribution and equal variance were expressed as mean ± standard deviation (SD) and compared between groups using the independent-samples *t*-test. Non-normally distributed variables were presented as median values and compared using the Wilcoxon rank-sum test. Categorical variables were expressed as frequency and percentage, and group differences were examined using the chi-square test or Fisher’s exact test.

A binary logistic regression analysis was performed to assess associations between patient characteristics and perioperative complications. Variables with significant differences in the univariate analysis, as well as those identified in prior research as potential confounders, were included in multivariate models. A multiple linear regression analysis was performed to further evaluate factors influencing postoperative endometrial thickness. All statistical tests were one-sided, and a *p*-value of < 0.05 was considered statistically significant.

## Results

**Figure 1:**
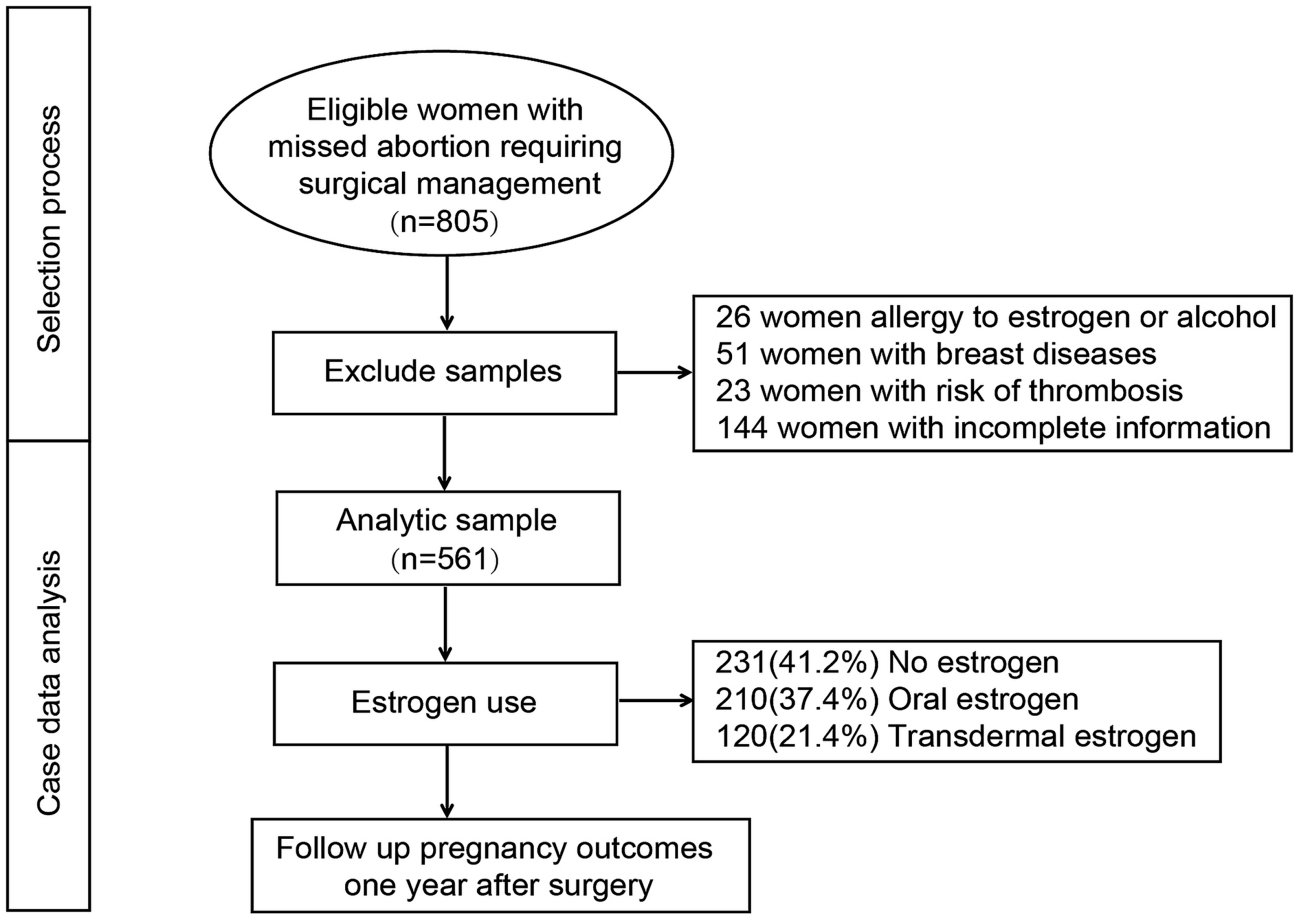
Selection process for this study.

The patient selection and exclusion process is illustrated in Figure 1. A total of 805 women with missed abortion who required surgical management were initially screened. After excluding those with allergies to estrogen or alcohol (*n* = 26), breast diseases (*n* = 51), risk of thrombosis (*n* = 23), and incomplete information (*n* = 144), 561 patients were included in the final analysis. The mean age was 31.02 ± 4.96 years, with a mean body mass index (BMI) of 21.88 ± 3.04 kg/m^2^. On average, patients had undergone 2.08 ± 1.26 prior to dilation and curettage (D&C) procedures, and 5.5% (*n* = 31) required emergency surgery. With regard to surgical type, 31.2% (*n* = 175) underwent ultrasound-guided abortion, while 68.8% (*n* = 386) underwent hysteroscopic abortion. Postoperatively, 41.2% (*n* = 231) did not receive estrogen therapy, 37.4% (*n* = 210) received oral estrogen, and 21.4% (*n* = 120) were treated with transdermal estrogen.

Baseline parameters, including age, BMI, duration of amenorrhea, maximum embryonic diameter, preoperative human chorionic gonadotropin (HCG), gravidity, parity, number of prior D&C procedures, emergency surgery rate, and type of surgical procedure, were comparable among the three groups (all *p* > 0.05) (Table 1). Operative outcomes, such as surgical duration and intraoperative blood loss, also showed no significant differences. Moreover, the incidence of surgical conversion, retained products of conception, and overall perioperative complications was low and similar across groups (all *p* > 0.05).

**Table 1:** Description of the patient perioperative characteristics by estrogen use.

Variables	No estrogen	Oral estrogen	Transdermal estrogen	*p*-value
Patients	*N* = 231	*N* = 210	*N* = 120	
Age (year)	30.80 ± 5.15	30.55 ± 4.77	30.36 ± 4.74	0.710^a^
BMI (kg/m^2^)	21.73 ± 3.37	22.23 ± 2.82	21.57 ± 2.74	0.102^a^
Duration of amenorrhea (week)	8.44 ± 1.19	8.56 ± 1.17	8.74 ± 1.48	0.113^a^
Max diameter of embryo (cm)	1.89 ± 1.16	1.95 ± 0.93	1.97 ± 0.84	0.742^a^
Preoperative HCG (IU/ml)	28.61 ± 2.45	29.07 ± 2.89	29.31 ± 3.50	0.066^a^
Gravidity	2.94 ± 1.69	2.72 ± 1.6	2.68 ± 1.63	0.238^a^
Parity	1 (1)	0 (1)	1 (1)	0.431^c^
Number of D&C abortions	2.11 ± 1.32	2.10 ± 1.2	1.98 ± 1.24	0.636^a^
Emergency surgery	16 (6.9%)	9 (4.3%)	6 (5.0%)	0.461^b^
Surgical type				0.377^b^
Ultrasound-guided abortion	65 (28.1%)	72 (34.3%)	38 (31.7%)	
Hysteroscopic abortion	166 (71.9%)	138 (65.7%)	82 (68.3%)	
Operative information				
Duration of surgery (min)	27.44 ± 19.37	26.58 ± 22.01	26.44 ± 19.6	0.872^a^
Bleeding volume (ml)	23.41 ± 39.94	32.53 ± 63.63	26.70 ± 66.18	0.227^a^
Uterine cavity adhesion	27 (11.7%)	56 (26.7%)	26 (21.7%)	<0.001^b^
Surgical conversion	2 (0.9%)	3 (1.4%)	1 (0.8%)	0.815^d^
Postoperative information				
Menstruation (day)	33.89 ± 4.22	34.06 ± 4.59	33.79 ± 4.93	0.858^a^
Retained products of conception	6 (2.6%)	7 (3.3%)	4 (3.3%)	0.882^d^
Perioperative complications	10 (4.3%)	12 (5.7%)	7 (5.8%)	0.753^d^

BMI, body mass index; HCG, human chorionic gonadotropin; D&C, Dilation and Curettage.

a Average and standard deviation. One-way analysis of variance.

b Number (percentage). Chi-squared test.

c Median (interquartile range). Welch’s test.

d Number (percentage). Fisher’s exact test.

A binary logistic regression analysis revealed the association between patient characteristics and perioperative complications, which identified a longer duration of amenorrhea (odds ratio [OR] = 1.18, 95% confidence interval [CI]: 1.08–1.27, *p* < 0.001), a larger embryonic diameter (OR = 1.25, 95% CI: 1.10–1.36, *p* = 0.013), higher preoperative HCG levels (OR = 1.02, 95% CI: 1.01–1.03, *p* = 0.038), and emergency surgery (OR = 1.83, 95% CI: 1.27–2.63, *p* = 0.002) as significant independent predictors of complications (Table 2). Uterine cavity adhesion was also an independent risk factor (OR = 1.65, 95% CI: 1.25–2.27, *p* = 0.024). Other variables, including age, BMI, prior number of D&C abortions, surgical type, and route of estrogen administration, were not significantly associated (all *p* > 0.05).

**Table 2:** Description of the patient’s postoperative endometrial and long-term pregnancy outcomes by estrogen use.

Variables	No estrogen	Oral estrogen	Transdermal estrogen	*p*-value
Postoperative condition	*N* = 231	*N* = 210	*N* = 120	
Endometrial thickness				
One month after surgery	4.59 ± 2.77	5.44 ± 2.73	5.70 ± 2.93	<0.001^a^
Three months after surgery	6.62 ± 2.60	7.07 ± 2.37	7.62 ± 2.30	0.001^a^
Uterine cavity adhesion	52 (22.5%)	32 (15.2%)	14 (11.7%)	0.022^b^
Adverse reactions during medication				
Breast tenderness	2 (0.9%)	23 (11%)	4 (3.3%)	<0.001^c^
Irregular vaginal bleeding	58 (25.1%)	48 (22.9%)	18 (15.0%)	0.092^b^
Skin acne/allergic rash	4 (1.7%)	12 (5.7%)	8 (6.7%)	0.041^c^
Systemic symptoms (headache, nausea, edema)	8 (3.5%)	24 (11.4%)	6 (5.0%)	0.003^c^
Medication duration (week)	–	6.39 ± 2.56	9.60 ± 2.70	<0.001^d^
Medication adherence	–	98 (46.7%)	82 (68.3%)	<0.001^b^
Pregnancy outcomes one year after surgery				0.819^c^
Pregnancy status	156 (67.5%)	144 (68.6%)	85 (70.8%)	
Spontaneous/missed abortion	33 (14.3%)	29 (13.8%)	18 (15%)	
Infertility	17 (7.4%)	15 (7.1%)	8 (6.7%)	
Contraception	25 (10.8%)	22 (10.5%)	9 (7.5%)	

BMI, body mass index; HCG, human chorionic gonadotropin; D&C, Dilation and Curettage.

a Average and standard deviation. One-way analysis of variance.

b Number (percentage). Chi-squared test.

c Number (percentage). Fisher’s exact test.

d Average and standard deviation. Student’s *t*-test.

Subgroup analysis revealed that, among the 29 patients with perioperative complications, 17 (58.6%) cases involved retained products of conception—14 of which occurred after ultrasound-guided abortion—while 4 cases of uterine perforation all occurred in hysteroscopic procedures. The remaining complications included intraoperative hemorrhage (*n* = 5), hysteroscopic fluid overload (*n* = 1), and postoperative infection (*n* = 2).

Table 3 outlines postoperative endometrial recovery, medication-related adverse reactions, and 1-year pregnancy outcomes. Endometrial thickness was significantly greater in both oral and transdermal estrogen groups compared with the no-estrogen group at 1 month (*p* < 0.001) and 3 months (*p* = 0.001) after surgery, with the transdermal group showing the highest values. Uterine cavity adhesion was less common among estrogen users compared with non-users (*p* = 0.022). Breast tenderness (11.0%) and systemic symptoms such as headache, nausea, or edema (11.4%) were more frequent in the oral group, whereas skin acne or allergic rash occurred in both oral (5.7%) and transdermal (6.7%) groups (*p* = 0.041). The mean duration of medication use was longer in the transdermal group (9.60 ± 2.70 weeks vs. 6.39 ± 2.56 weeks, *p* < 0.001), and adherence was significantly higher compared with the oral group (68.3% vs. 46.7%, *p* < 0.001). At 1-year follow-up, pregnancy outcomes—including conception rates, spontaneous or missed abortion, infertility, and contraception use—did not differ significantly among groups (*p* = 0.819), although higher conception rates were observed in the estrogen groups, particularly in the transdermal group (70.8%).

**Table 3:** Association between patient characteristics and perioperative complications.

Variables	Exp(B)	95% CI	*p*-value
Age (year)	1.01	(0.97,1.04)	0.541
BMI (kg/m^2^)	0.98	(0.86,1.13)	0.697
Duration of amenorrhea (week)	1.18	(1.08,1.27)	<0.001
Max diameter of embryo (cm)	1.25	(1.10,1.36)	0.013
Preoperative HCG (IU/ml)	1.02	(1.01,1.03)	0.038
Number of D&C abortions	1.20	(0.69,1.58)	0.750
Emergency surgery	1.83	(1.27,2.63)	0.002
Surgical type (Ultrasound/hysteroscopic)	0.91	(0.65,1.26)	0.679
Estrogen use (No/Yes)	1.41	(0.71,1.91)	0.277
Estrogen administration methods (Oral/transdermal)	1.47	(0.72,1.99)	0.142
Uterine cavity adhesion	1.65	(1.25,2.27)	0.024

BMI, body mass index; HCG, human chorionic gonadotropin; D&C, Dilation and Curettage.

**Figure 2:**
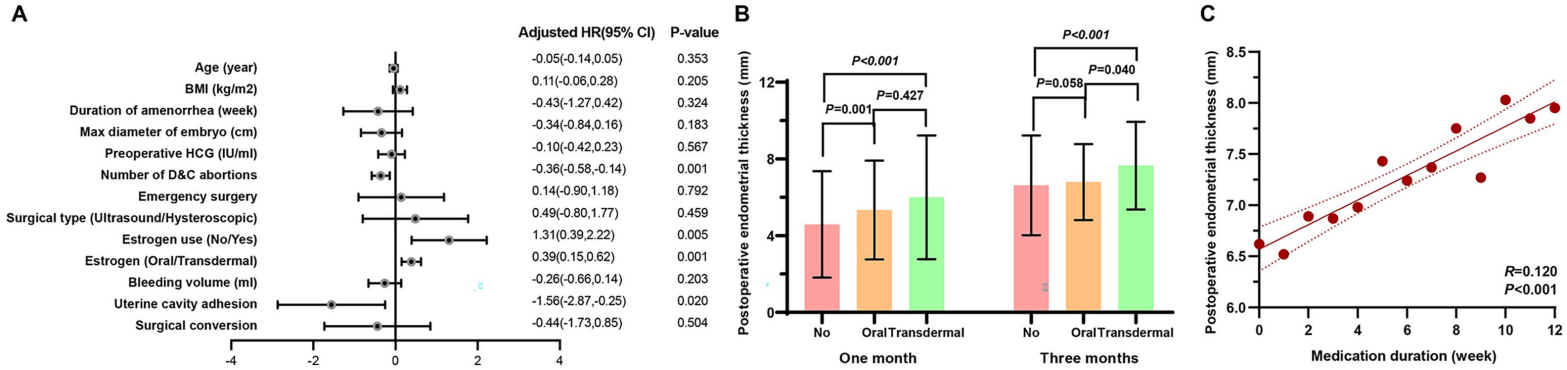
Associations between estrogen therapy and postoperative endometrial thickness. **(A)** Multivariate analysis of factors associated with postoperative endometrial thickness. Estrogen use was independently correlated with greater endometrial thickness, with transdermal administration showing a significant positive effect; **(B)** Comparison of endometrial thickness among the no-estrogen, oral estrogen, and transdermal estrogen groups at 1 and 3 months after surgery. Both estrogen groups demonstrated significantly greater thickness compared with the no-estrogen group. At 3 months, the transdermal group showed significantly higher values than the oral group; **(C)** Correlation between duration of estrogen therapy and endometrial thickness at 3 months postoperatively. A positive linear association was observed, indicating that longer estrogen treatment was related to greater endometrial thickness.

The multiple linear regression analysis was employed to further analyze the factors influencing postoperative endometrial thickness. Estrogen use was independently associated with greater endometrial thickness (Beta = 1.31, 95% CI: 0.39–2.22, *p* = 0.005), with a significant positive effect for transdermal administration (Beta = 0.39, 95% CI: 0.15–0.62, *p* = 0.001). In addition, intrauterine adhesion was associated with a 1.56-mm reduction in endometrial thickness at 3 months postoperatively (Beta = −1.56, 95% CI: −2.87 to −0.25, *p* = 0.020) (Figure 2A).

Other baseline and perioperative factors, including age, BMI, amenorrhea duration, embryo diameter, preoperative HCG, number of prior D&C abortions, surgical type, and bleeding volume, were not significantly associated. Figure 2B illustrates group comparisons of endometrial thickness at 1 and 3 months. Both oral and transdermal groups had significantly greater thickness than the no-estrogen group (all *p* ≤ 0.001), with the transdermal group maintaining significantly greater thickness than the oral group at 3 months (*p* = 0.040). Figure 2C demonstrates a positive linear correlation between medication duration and endometrial thickness at 3 months postoperatively. Within 3 months after surgery, each additional week of estrogen use was associated with an average increase of 0.12 mm in endometrial thickness (R = 0.120, *p* < 0.001).

## Discussion

In this retrospective longitudinal cohort study, we evaluated the effects of different postoperative estrogen regimens on endometrial recovery and long-term reproductive outcomes in women with missed abortion. Our results demonstrated that both oral and transdermal estrogen were significantly associated with improved postoperative endometrial thickness and a reduced proportion of women with decreased menstrual flow compared with those in the no-estrogen group. Notably, transdermal estrogen showed superior tolerability and adherence while maintaining comparable efficacy to oral estrogen. At 1-year follow-up, although no significant differences were detected in overall pregnancy outcomes among the three groups, numerically higher pregnancy rates were observed in the estrogen-treated groups, particularly with transdermal administration.

Our findings are consistent with previous evidence suggesting that estrogen therapy facilitates endometrial regeneration after intrauterine surgery by promoting epithelial proliferation and angiogenesis (16). Our findings are consistent with recent research demonstrating that postoperative estrogen therapy facilitates endometrial regeneration and significantly reduces intrauterine adhesion formation after uterine evacuation (15, 17). Prior randomized trials and observational studies have reported improvements in endometrial thickness and reduced intrauterine adhesion rates with estrogen use; however, a majority of them have focused on short-term outcomes. Few studies have compared oral and transdermal estrogen administration (18, 19). In line with pharmacological data, our study observed a better side-effect profile and greater patient adherence with transdermal estrogen, supporting its use as a preferable alternative in postoperative management. Similarly, a multicenter cohort study showed that transdermal estrogen was associated with improved patient adherence and fewer systemic side effects compared with oral estrogen, supporting our observation of better tolerability and compliance with the transdermal regimen (20).

The clinical relevance of these findings lies in guiding optimized postoperative hormonal therapy strategies to preserve fertility in women with missed abortion. Oral estrogen, while effective, carries risks associated with hepatic first-pass metabolism, including breast tenderness, irregular bleeding, and systemic side effects (4). Transdermal estrogen avoids hepatic metabolism and provides more stable serum concentrations, which may explain its superior tolerability and adherence in our cohort. From a reproductive perspective, both routes appeared effective in promoting endometrial repair, but the higher adherence and comparable efficacy of transdermal estrogen make it a more favorable option in clinical practice (6).

Moreover, in line with recent evidence, although short-term endometrial recovery differed among treatment groups, long-term conception and live birth rates showed no statistically significant differences, suggesting that estrogen primarily exerts short- to mid-term benefits on endometrial repair rather than direct effects on fertility outcomes (15, 21).

Beyond estrogen use, our regression analyses identified longer amenorrhea duration, larger embryonic diameter, elevated preoperative HCG levels, and emergency surgery as significant predictors of perioperative complications (22). Furthermore, intrauterine adhesion was independently associated with decreased endometrial thickness at 3 months postoperatively, underscoring the importance of early prevention and intervention (23, 24). These results emphasize that, in addition to pharmacologic support, careful surgical technique and early detection of adhesions remain critical to optimizing outcomes.

The strengths of this study include its longitudinal design, relatively large sample size, and comprehensive follow-up covering both short-term and long-term outcomes. Nevertheless, several limitations must be acknowledged. First, as a retrospective single-center study, inherent selection bias and unmeasured confounding cannot be fully excluded. Second, medication allocation was not strictly randomized, and patient preferences may have influenced treatment adherence. Third, pregnancy outcomes at 1 year may not fully capture long-term reproductive potential, and larger prospective studies with live birth outcomes are warranted. Finally, although the sample size of this study was sufficient based on power calculation, larger multicenter studies are warranted to validate and strengthen the generalizability of our results.

## Conclusion

This study demonstrates that postoperative estrogen therapy improves endometrial recovery in women undergoing surgical evacuation for missed abortion. Both oral and transdermal estrogen are effective, but transdermal estrogen administration provides superior tolerability and adherence. While long-term pregnancy outcomes did not differ significantly, higher conception rates were observed in the estrogen-treated groups. These findings support the clinical use of transdermal estrogen as a favorable therapeutic strategy for promoting endometrial repair and fertility preservation after missed abortion. Future prospective randomized trials are needed to confirm these results and to establish standardized guidelines for postoperative hormonal management.

## Data Availability

The original contributions presented in the study are included in the article/supplementary material, further inquiries can be directed to the corresponding authors.
